# Lynch syndrome and colorectal cancer: A review of current perspectives in molecular genetics and clinical strategies

**DOI:** 10.32604/or.2025.063951

**Published:** 2025-06-26

**Authors:** RAQUEL GÓMEZ-MOLINA, RAQUEL MARTÍNEZ, MIGUEL SUÁREZ, ANA PEÑA-CABIA, MARíA CONCEPCIóN CALDERÓN, JORGE MATEO

**Affiliations:** 1 Department of Laboratory Medicine, Virgen de la Luz Hospital, Cuenca, 16002, Spain; 2 Gastroenterology Department, Virgen de la Luz Hospital, Cuenca, 16002, Spain; 3 Medical Analysis Expert Group, Institute of Technology, Universidad de Castilla-La Mancha, Cuenca, 16071, Spain; 4 Medical Analysis Expert Group, Instituto de Investigación Sanitaria de Castilla-La Mancha (IDISCAM), Toledo, 45071, Spain

**Keywords:** Lynch Syndrome (LS), Colorectal Cancer (CRC), Hereditary Nonpolyposis Colorectal Cancer (HNPCC), Genetic testing, DNA Mismatch Repair (MMR), Endoscopy, Colonoscopy, Genetic counseling

## Abstract

Lynch syndrome (LS), also known as hereditary non-polyposis colorectal cancer (HNPCC), is an inherited condition associated with a higher risk of colorectal cancer (CRC) and other cancers. It is caused by germline mutations in DNA mismatch repair (MMR) genes, including *MLH1, MSH2, MSH6* and *PMS2*. These mutations lead to microsatellite instability (MSI) and defective DNA repair mechanisms, resulting in increased cancer risk. Early detection of LS is crucial for effective management and cancer prevention. Endoscopic surveillance, particularly regular colonoscopy, is recommended for individuals with LS to detect CRC at early stages. Additionally, universal screening of CRC for MMR deficiency can help identify at-risk individuals. Genetic counseling plays a valuable role in LS by guiding patients and their families in understanding the genetic basis, making informed decisions regarding surveillance and prevention, and offering reproductive options to reduce the transmission of pathogenic variants of the offspring. The aim of this review is to outline current strategies for the diagnosis, surveillance, and management of LS, with a focus on the role of genetic counseling, endoscopic screening, and emerging therapeutic approaches to mitigate cancer risk in affected individuals.

## Introduction

Lynch Syndrome (LS) is associated with a significantly increased lifetime risk of developing colorectal cancer (CRC), with estimates ranging from 30% to 74% depending on germline pathogenic variant [1–3]. LS is the most common heritable CRC syndrome, with an estimated prevalence of 1 in 440 in the general population [4,5]. The syndrome also increases the risk of several other malignancies, such as those of the cancer of stomach, small intestine, pancreas, biliary tract, urinary tract, brain and skin [2,4,6]. Women with LS have a particularly high risk of endometrial and ovarian cancers [7]. Approximately 20% of CRC patients have at least one first-degree relative with a history of this cancer [8,9].

It is recommended that individuals with LS undergo regular surveillance, including colonoscopy every 1–2 years starting in their twenties, to detect and manage CRC early [10,11]. Genetic testing and family history are essential for diagnosing LS and universal genetic testing of colorectal cancers is increasingly used to identify high-risk individuals [12]. Genetic counseling offers additional benefits by helping patients and their families make informed decisions, providing tailored recommendations for personalized management, surveillance and prevention strategies, and presenting reproductive options to reduce the transmission of pathogenic variants to future generations [13].

## Molecular Genetics

LS or hereditary nonpolyposis colorectal cancer (HNPCC) is the most common hereditary CRC, accounting for 2%–4% of cases [9]. It is an autosomal dominant disorder caused by germline variants in DNA MMR genes [14]. MMR dysfunction leads to replication errors, particularly in microsatellites, resulting in microsatellite instability (MSI) or loss of MMR protein expression, the hallmark of LS [15]. Advances in epidemiology and molecular insights, aided by multigene panel testing, have enhanced strategies for cancer prevention, risk reduction and immunotherapy [16].

### DNA mismatch repair mechanism

**Figure 1:**
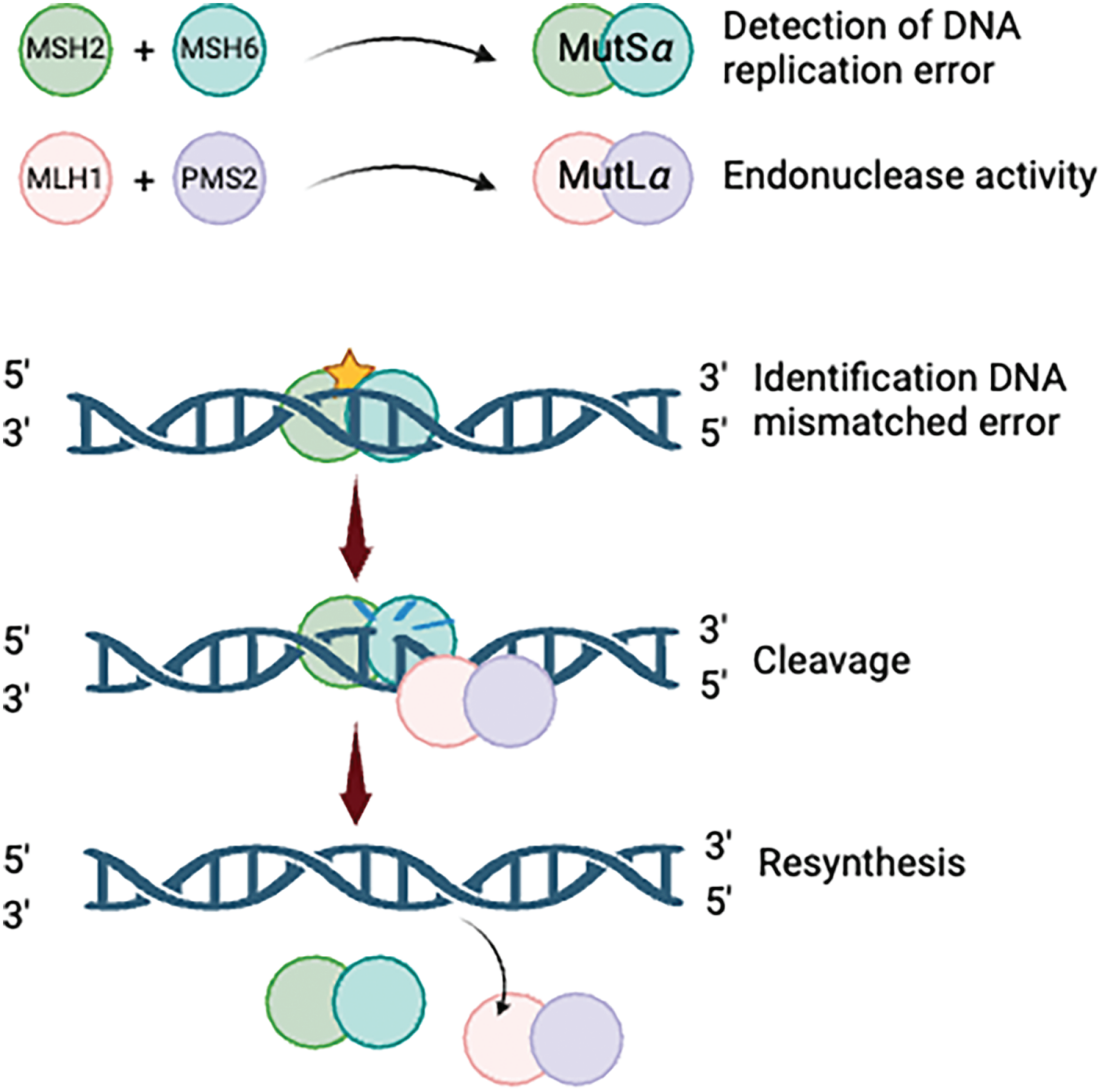
The process of DNA mismatch repair. Created with BioRender.com.

The DNA mismatch repair (MMR) system is crucial for maintaining genomic stability by correcting base substitution errors and small insertion-deletion mismatches that arise during DNA replication [17]. This highly regulated process relies on the coordinated activity of specialized protein complexes. Mismatch recognition is performed by two heterodimeric protein complexes: *MutSα (MSH2* and *MSH6*), which efficiently detects single base-pair mismatches and small insertion-deletion loops, and *MutSβ (MSH2* and *MSH3*), which targets larger insertion-deletion loops. Once mismatches are identified, downstream repair is facilitated by MutL complexes, including *MutLα* (*MLH1* and *PMS2*), which functions as a key endonuclease in the repair process. *MutLβ (MLH1* and *PMS1*) and *MutLγ (MLH1* and *MLH3*) further assist in excision and resynthesis, ensuring accurate DNA replication [18,19]. These interactions collectively uphold genomic fidelity and prevent mutational accumulation (Fig. 1).

Germline variants in MMR genes follow an autosomal dominant inheritance pattern, where a single mutated allele is inherited. However, defective MMR function requires biallelic inactivation, typically seen in LS, where a germline variant in one MMR allele is accompanied by somatic inactivation of the second allele [20]. This somatic inactivation may occur through various mechanisms, including mutation, loss of heterozygosity, or epigenetic silencing via promoter hypermethylation. The resulting biallelic inactivation of MMR genes causes a deficient MMR system, leading to an elevated mutation rate and genomic instability due to the inability to repair DNA mismatches during replication [21].

These mismatches often occur in microsatellites, regions of repetitive nucleotide sequences. Tumors with defective MMR frequently show MSI, a defining feature of Lynch-like syndrome (LLS). MSI can affect genes regulating key cellular processes, such as cell growth (e.g., *TGF-beta, insulin-like growth factor receptors*), apoptosis (e.g., *Caspase 5, Bax*) and even MMR genes themselves (e.g., *hMSH3, hMSH6)* [22]. Accumulation of variants in these critical genes is thought to drive tumorigenesis, underscoring the crucial role of MMR deficiency in LLS [23].

Deletions in the terminal codon of the *EPCAM* gene, located upstream of *MSH2*, can result in epigenetic silencing of *MSH2*, particularly in tissues where *EPCAM* is expressed [24]. When the deletion is confined to the *EPCAM* stop codon, the phenotype is typically restricted to CRC. However, deletions that extend into the *MSH2* promoter region lead to a more comprehensive LS phenotype, which encompasses a broader spectrum of cancers [25]. Although *EPCAM* is not classified as an MMR gene, its deletion can contribute to LS by silencing the adjacent *MSH2* gene.

### Phenotypic correlations of molecular alterations

Pathogenic variants in MMR genes result in impaired DNA repair, leading to an increased mutation rate and, consequently, heightened cancer risk. The cancer risk and spectrum of associated malignancies in LS are known to vary based on the specific MMR gene involved. Increasing evidence suggests that the MMR gene affected may influence the molecular pathogenesis of CRC within the context of LS [22,24]. The role of specific MMR gene variants (p*ath_MMR*) in cancer penetrance is still debated [25]. In this regard, no significant differences in penetrance have been observed between missense and truncating variants in individuals carrying pathogenic *MLH1* (*path_MLH1*) or *MSH2* (*path_MSH2*) variants [26].

Mutations in MMR genes are primarily missense or truncating, with missense and truncating variants constituting the majority of pathogenic variants in *MLH1* (40% each), *MSH2* (31% and 49%, respectively) and *MSH6* (49% and 43%, respectively). In contrast, *PMS2* mutations are predominantly missense (62%), with truncating variants occurring less frequently (24%) [27]. These findings underscore the need for further investigation into the relationship between specific mutation types and cancer penetrance. Data from the International Mismatch Repair Consortium (IMRC) show significant variability in CRC risk among carriers of different MMR gene variants [28]. The risk is influenced by factors such as gene, sex, and geographic region. The penetrance of CRC in LS carriers ranges widely: some individuals have a relatively low risk, with less than 20% developing CRC, while others may have a much higher risk, with over 80% developing the disease. A significant proportion, between 10%–19%, have a moderate risk, with a 40%–60% chance of developing CRC. This highlights the variability in cancer risk, which can depend on factors such as the specific MMR gene mutation and other genetic or environmental factors.

The variable penetrance and expressivity of LS suggest the existence of four distinct inherited syndromes, each associated with a specific MMR gene. According to the Online Mendelian Inheritance in Man (OMIM) database, these four syndromes correspond to pathogenic variants in *MLH1*, *MSH2, MSH6* and *PMS2*, while 3′ *EPCAM* deletions are recognized as an alternative mechanism leading to *MSH2* silencing [29]. The International Society for Gastrointestinal Hereditary Tumours (InSiGHT) uses its international database to classify MMR gene variants as pathogenic or non-pathogenic, while the Prospective Lynch Syndrome Database (PLSD) provides data on penetrance and expressivity of pathogenic variants to guide patient counseling [30,31].

Recent analyses highlight distinct cancer risk profiles among *path_MMR* carriers (Table 1). Møller et al. compared CRC incidences in *path_MMR* either in the PLSD and IMRC. In the PLSD, 8.153 carriers were followed under colonoscopic surveillance, identifying 578 CRC cases. By age 70, cumulative CRC incidences were 52% for males and 41% for females with *path_MLH1*, 50% and 39% for *path_MSH2*, 13% and 17% for *path_MSH6*, and 11% and 8% for *path_PMS2*. In contrast, the IMRC cohort reported lower incidences: 40% and 27% for *path_MLH1*, 34% and 23% for *path_MSH2*, 16% and 8% for *path_MSH6*, and 7% and 6% for *path_PMS2* [32]. These findings underscore the heterogeneity in CRC risk, particularly between *MLH1/MSH2* and *MSH6/PMS2* carriers.

**Table 1:** Cumulative risk and mortality of colorectal cancer

	Møller et al. [33]		Dominguez-Valentin et al. [34]			
	**Cumulative incidence at 70 years**		**Cumulative incidence at 65 years**		**Mortality**	
*path_MMR*	Male	Female	Male	Female	Male	Female
*path_MLH1*	52%	41%	48%	36%	6%	5%
*path_MSH2*	50%	39%	42%	30%	5%	4%
*path_MSH6*	13%	17%	13%	10%	2 %	1%
*path_PMS2*	11%	8%	10%	3%	1%	0%

Additionally, age and sex significantly influence CRC risk among *path_MMR* carriers, with particularly wide confidence intervals for *MSH6* and *PMS2* [33,34]. For CRC, cumulative lifetime risks by age 75 are estimated at 46% for *path_MLH1*, 43% for *path_MSH2*, and 15% for *path_MSH6* carriers, with *MLH1* and *MSH2* carriers showing significantly higher risks (40%–70%) compared to *MSH6* and *PMS2* heterozygotes (10%–20%) [35]. Comparing just the European carriers in the two series gave similar findings [36]. It is essential to highlight that these incidences are derived from patients undergoing regular colonoscopy surveillance. As reported by Dominguez-Valentin et al., LS-associated cancers outside the colorectum, particularly in women and among *path_MSH2* carriers, substantially contribute to the overall cancer burden [37]. Furthermore, familial risk factors contribute to substantial within-gene variation in CRC risk with and without colonoscopy surveillance, underscoring the importance of personalized risk assessment for precision prevention and early detection of CRC in *path_MMR* carriers [28,35,38].

### Genetic diagnosis of LYNCH syndrome

The diagnosis of LS involves a combination of tumor screening and genetic testing. Multiple clinical guidelines recommend universal screening for MMR deficiency, particularly in new CRC cases, to identify potential LS cases for subsequent constitutional testing [39–41]. Universal screening is a cost-effective approach for LS detection and has significant implications for cancer management, particularly with the advent of checkpoint inhibition therapies [42].

Tumor screening methods typically include immunohistochemical analysis (IHC) for MMR protein expression and MSI testing. Both methods exhibit similar sensitivity and specificity, although neither is entirely accurate [43]. Some LS-related variants, such as certain missense or truncating variants, may result in stable but nonfunctional proteins, which can lead to false-negative results. Moreover, clonal heterogeneity or low tumor purity may cause MSI-positive tumors to show normal MMR protein expression. IHC offers the additional benefit of identifying specific MMR proteins with abnormal expression, allowing for the identification of the gene likely altered constitutionally. For example, the loss of both *MSH2* and *MSH6* proteins points to an *MSH2* alteration, while the loss of *MLH1* and *PMS2* indicates an *MLH1* variant. However, exceptions to this pattern exist, so constitutional testing should not be limited to the gene identified by IHC [44].

Regarding IHC testing, the absence of antibodies for either four or two MMR proteins has been investigated to optimize the detection of MMR deficiencies. The four-stain MMR IHC method evaluates the expression of *MLH1, MSH2, MSH6*, and *PMS2* proteins, allowing for comprehensive detection of MMR deficiencies. This approach identifies variants in any of the four key MMR genes, ensuring accurate LS screening. In contrast, the two-stain method assesses only *MSH6* and *PMS2*, relying on the fact that *MLH1* and *MSH2* deficiencies typically lead to secondary loss of *PMS2* and *MSH6*, respectively. However, this method may fail to detect certain *MSH2* variants, as some tumors with *MSH2* loss can retain *MSH6* expression, increasing the risk of false-negative results. While the two-stain approach offers potential cost and time savings, the four-stain method remains the most reliable for minimizing diagnostic errors [45–47].

Next-generation sequencing (NGS) has emerged as a valuable tool for detecting variants associated with LS. Unlike traditional methods, NGS allows for comprehensive analysis of multiple genes involved in MMR, improving sensitivity and accuracy in identifying pathogenic variants [48,49]. By sequencing tumor DNA, NGS can detect both germline and somatic variants, including double somatic mutations, which are characteristic of LLS [50,51]. This approach not only enhances the detection of LS-related variants but also provides valuable information for treatment planning, such as identifying actionable variants in other genes like *KRAS, NRAS* or *BRAF* [52]. By enabling more detailed and efficient genetic testing, NGS plays a crucial role in refining LS diagnosis and guiding clinical management.

In addition, NGS can also provide valuable information on tumor mutation burden (TMB), a key biomarker for predicting response to immunotherapy. TMB, assessed through NGS panels, is associated with better outcomes in metastatic colorectal cancer (mCRC) and can help identify patients likely to benefit from immune checkpoint inhibitors (ICIs). Antoniotti et al. [53] demonstrated that TMB can stratify mCRC patients for ICI response, while Xiao et al. [54] emphasized combining TMB with MSI status for more accurate predictions. Additionally, Schrock et al. [55] and Manca et al. [56] highlighted TMB’s predictive value in MSI-high mCRC, with higher TMB correlating with improved progression-free survival. The National Comprehensive Cancer Network (NCCN) recognizes TMB as a potential biomarker for ICI response, particularly for pembrolizumab in TMB-high tumors [57]. Overall, NGS-based TMB testing aids in personalized treatment strategies for CRC.

The use of tumor mutational signatures for enhanced LS detection through NGS is increasingly common, even in diagnostic settings, and is becoming more cost-effective. Recent advancements in NGS technology have allowed for the comprehensive analysis of tumor mutational signatures, which improves LS detection by identifying both germline and somatic variants in MMR genes. For instance, the TumorNext-Lynch-MMR [58] panel provides a detailed genetic profile, including MSI status and loss of heterozygosity. Furthermore, the clinical utility of NGS has been demonstrated in molecular subtyping and LS screening, especially in endometrial cancer, highlighting NGS’s ability to reduce missed diagnoses [59]. With the decreasing cost of NGS, it is becoming more accessible for broader clinical use [60].

However, despite these advances, NGS-based tumor mutational signature analysis remains outside of routine diagnostic procedures and recommended guidelines. Although the American Society of Clinical Oncology (ASCO) and other expert consensus groups recognize the potential of NGS for determining MSI/MMR status and TMB, they recommend its application primarily in specialized centers or through validated central laboratory methods [61,62].

## Clinical Management and Surveillance Strategies in Lynch Syndrome

Colonoscopy and its follow-up have proven to be a cost-effective measure for reducing incidence, mortality, and improving quality of life [63]. Adherence to the surveillance program is crucial to achieving these objectives, as well as the early identification of patients to begin colonoscopies as soon as possible.

As previously mentioned, not all pathological variables present the same cancer risk over a lifetime. It is estimated that patients with a *MLH1* pathogenic variant have a risk close to 46%, and those with *MSH2*, a 35% risk. Moreover, patients carrying *MSH6* and PMS2 pathogenic variants have a 20% and 10% risk, respectively [33]. These data suggest that the initiation and follow-up of colonoscopies should vary based on the patient’s genetic pathogenic variant. Patients with *MLH1* and *MSH2* pathogenic variants should start surveillance at age 25, while those with *MSH6* and *PMS2* pathogenic variants should start at age 35 [37,64,65].

Surveillance of these patients is not uniform and varies according to different clinical guidelines. However, there is consensus across almost all guidelines that surveillance should occur every 1 to 3 years [66,67]. The differences between guidelines stem from the fact that most studies used to determine these surveillance intervals are retrospective, and prospective data are lacking. Thus, each guideline adapts the available evidence to its respective population. The scientific evidence clearly shows that intervals longer than 3 years increase the risk of CRC development, and intervals shorter than 1 year do not show any benefit in detecting advanced lesions or CRC [66–69].

Surveillance in patients with the *PMS2* pathogenic variants may deviate from this 1–3 years interval. Due to its low penetrance, the risk of developing CRC in these patients is lower compared to other MMR variants [34,63]. Consequently, the latest European guidelines recommend extending screening colonoscopies to every 5 years [37]. This suggests that further studies are needed in LS patients to establish more personalized surveillance, thus avoiding unnecessary invasive testing.

A frequently aspect overlooked when reviewing clinical guidelines for LS patient surveillance is that these recommendations are intended for asymptomatic patients. Patients with a diagnosis of LS and symptoms suggestive of malignancy, such as new-onset abdominal pain, constitutional syndrome, anemia, or rectal bleeding, require proper evaluation and colonoscopy before the established surveillance interval. In fact, although based on low-level evidence, European guidelines recommend performing a colonoscopy as soon as possible [51].

### Colonoscopy quality and technique in lynch syndrome management

Colonoscopy quality is crucial for lesion detection in the general population and is even more critical in LS patients. Adenomas in LS tend to be located proximally, have a flat morphology, and often show high-grade dysplasia, even when smaller than 10 mm, making them more challenging to detect [70,71]. The key indicators of colonoscopy quality include:

– Adequate preparation: defined as a Boston Bowel Preparation Scale score ≥ 6, with no segment scoring ≤ 1.
– Complete colonoscopy or cecal pole intubation.
– Adenoma detection rate (ADR): should exceed 30% for expert endoscopists [72].
– Complete resection of adenomas/polyp removal, primarily through en-bloc resection with negative margins and favorable histology [73].

When these quality criteria are met, adenoma miss rate (AMR), and post-colonoscopy colorectal cancer (PCCRC) rates decrease. In LS patients, the AMR is higher than in the general population (over 48% *vs*. 25%), and the PCCRC rate approaches 8% after 10 years [74,75]. Even with strict adherence to these conditions, CRC may still occur due to new lesion development or rapid adenoma-carcinoma progression between screenings [76].

The use of chromoendoscopy has been debated for improving lesion detection, with earlier studies suggesting better detection of polyps in LS patients [77–79]. However, advances in high-definition (HD) endoscopes and white light endoscopy (WLE) have improved detection rates, and studies now demonstrate the non-inferiority of WLE over dye-based chromoendoscopy [80,81]. Clinical guidelines recommend HD endoscopy routinely, while chromoendoscopy is suggested only for endoscopists with lower adenoma detection rates or less experience [37,51].

Artificial intelligence (AI)-based systems such as computer-aided polyp detection (CADe) and diagnosis (CADx) have been explored. While AI has shown an increase in ADR in the general population, it has not demonstrated improvements in LS patients when using high-quality colonoscopies [82–84]. AI may be beneficial for endoscopists with lower ADR or less experience.

Perhaps the most important aspect, often overlooked, is patient awareness of their condition and cancer risk. Explaining the significance of LS, the necessity of surveillance, and the importance of high-quality colonoscopies is essential for reducing the risk of CRC. Non-adherence to screening is a major risk factor for CRC, especially in *MLH1* and *MSH2* variant carriers [67]. Adherence to the recommended screening intervals of 1–3 years, along with optimal bowel preparation, reduces this risk. In cases of suboptimal preparation, European guidelines recommend repeating the colonoscopy within three months [51].

In conclusion, high-quality colonoscopy, adherence to established standards, the use of the best available technology, and patient compliance with surveillance are the best strategies to minimize CRC risk. However, the possibility of CRC development between colonoscopies remains a concern, requiring continued research to better understand the underlying mechanisms [74].

### Prevention

Aspirin has shown benefits as a chemopreventive agent for reducing the risk of CRC in LS patients. The randomized CAPP2 trial demonstrated that a daily dose of 600 mg of aspirin for 2–4 years significantly reduced CRC incidence after 5 years of treatment initiation and was well tolerated. Furthermore, more recent studies suggest this protective effect extends up to 20 years post-treatment [85]. The exact mechanism by which aspirin reduces CRC risk remains unknown.

The optimal aspirin dose is under investigation in the ongoing CAPP3 trial. However, based on observational studies in the general population, a minimum dose of 75 mg is recommended, with adjustments based on body weight [37,86,87].

Lifestyle factors have not been clearly linked to an increased cancer risk in LS patients. Nevertheless, general health recommendations include regular physical activity to prevent sedentary behavior, maintaining a healthy body mass index, abstaining from smoking, and minimizing alcohol consumption [37,88–90].

### Surgery, chemotherapy and immune checkpoint inhibitors

Due to the high efficacy of quality colonoscopies, prophylactic surgery as primary prevention in the absence of lesions is not recommended for any genetic variants of LS [37]. For large lesions or early-stage tumors, endoscopic submucosal dissection (ESD) is the preferred first-line treatment. Although technically complex, ESD is a safe procedure that enables en-bloc resections, minimizes recurrence, and preserves the colonic surface, avoiding extensive resections in these patients [91]. Another endoscopic option is endoscopic full-thickness resection (eFTR), although it is limited to lesions measuring 2–3 cm [92].

In cases of invasive cancer, surgical intervention becomes necessary. For invasive CRC, extended surgeries may be considered based on individual patient factors rather than solely on MMR mutation status. Clinical guidelines recommend subtotal colectomy for *MLH1* and *MSH2* variant carriers due to a higher risk of metachronous CRC, whereas segmental resection is typically sufficient for *MSH6* and *PMS2* carriers [37]. However, recent studies suggest that *MLH1/MSH2* carriers may also be treated with traditional segmental surgeries, provided they undergo close post-surgical monitoring. This approach acknowledges a higher metachronous CRC risk but avoids the morbidity of extensive resections, as expanded surgeries have not demonstrated survival benefits [93,94]. All surgical options, including risks and benefits, should be thoroughly discussed with the patient, considering their comorbidities and preferences.

For rectal cancer in LS patients, standard surgical procedures such as anterior resection or abdominoperineal amputation, depending on tumor location, are recommended [37]. Extended surgeries should be reserved primarily for synchronous tumors. Creating an ileoanal pouch is particularly complex and should be performed in high-volume specialized centers [95].

In early-stage CRC, adjuvant chemotherapy with capecitabine or 5-fluorouracil (5-FU) does not improve overall survival and is associated with increased toxicity. These tumors typically exhibit mismatch repair deficiency (dMMR) and high microsatellite instability (MSI-H). This results in limited cytotoxicity of 5-FU, primarily due to alterations in the anabolic pathway mediated by thymidylate synthase (TS). Tumors associated with LS show high expression of this enzyme, the primary target of this chemotherapeutic agent, thereby reducing the sensitivity to 5-FU. Moreover, base excision repair and dMMR systems more effectively remove 5-FU from cellular DNA, further decreasing its cytotoxicity in these cases. Additionally, overexpression of resistance genes such as ABCC1 and ABCC5 may reduce intracellular accumulation of 5-FU [96–98]. These mechanisms collectively explain why these patients do not benefit from treatments typically used in CRC. Consequently, these patients have favorable prognoses with surgical management alone [99].

However, tumors that are proficient in mismatch repair (pMMR) or lack high microsatellite instability (MSS) should be considered for standard chemotherapeutic treatments due to their lower response to ICI, attributed to reduced immunogenicity. Additionally, LS-associated CRC with mucinous differentiation or signet-ring cells also exhibit resistance to ICI, suggesting a potentially greater benefit from chemotherapy [100]. For this reason, combinations of ICI and chemotherapy are being investigated in multiple clinical trials. These combinations have demonstrated improvements in response rates, progression-free survival (PFS) and overall survival (OS) compared to standard chemotherapy.

Although resistance to chemotherapy accounts for up to 80%–90% of therapeutic failures, some cytotoxic agents have shown the ability to enhance the efficacy of ICIs. These combinations have yielded higher response rates and better survival outcomes compared to chemotherapy monotherapy. It has been established that a resistance probability threshold of 5% is appropriate to define resistance to ICI-chemotherapy combinations, recognizing that the incidence of pseudoprogression may be lower when immunotherapy is administered alongside cytotoxic agents [101].

The advent of immunotherapy has revolutionized the treatment of CRC in the context of LS. While it is already considered the first-line treatment for metastatic cancer, its use in early-stage disease remains under investigation, with promising results [57,102]. The high immunogenicity of these tumors makes ICIs an excellent therapeutic option. In this setting, PD-1 inhibitors are employed, as these tumors exhibit a high density of T lymphocytes and a significant number of neoantigens. These agents block the interaction between the PD-1 receptor on T cells and its ligands PD-L1 and PD-L2 on tumor cells, thereby enabling cytotoxic T cells to recognize and destroy tumor cells [103].

Immunotherapy with ICIs has demonstrated high efficacy in the treatment of mCRC with dMMR or MSI-H. In advanced stages, trials such as CHECKMATE-142 have shown that the combination of nivolumab and ipilimumab achieves objective response rates (ORR) of up to 69%, with PFS in patients with dMMR mCRC and complete responses in 13% of cases. The combination of PD-1 inhibitors (nivolumab and pembrolizumab) with CTLA-4 inhibitors (ipilimumab) has demonstrated superior efficacy compared to PD-1 inhibitors alone [104]. Ongoing trials, such as COMMIT and CHECKMATE-8HW, aim to optimize combination therapies of ICIs and chemotherapy in treatment-naive patients with dMMR mCRC. However, a significant proportion of patients experience primary or secondary resistance to ICIs, highlighting the need for a deeper understanding of the molecular pathways underlying this resistance [105].

At early stages of CRC, immunotherapy has shown limited benefit, although several clinical trials are currently assessing its efficacy. The KEYNOTE-177 trial demonstrated that first-line pembrolizumab improves PFS in patients with dMMR CRC compared to conventional chemotherapy, despite its effectiveness in first stages remains uncertain [106]. Moreover, neoadjuvant research is gaining attention. Several clinical trials, such as the IMPOWER010 (NCT02486718) study, are exploring the use of ICIs in combination with surgery in patients with dMMR, based on the hypothesis that early activation of the immune system could reduce the risk of recurrence [107]. Preliminary results have shown significant tumor reductions and enhanced activation of immune responses in patients with CRC treated with this approach.

Neoadjuvant immunotherapy is being evaluated as a promising strategy for early-stage CRC. In this context, studies such as NICHE (NCT03026140) and VOLTAGE-A (NCT02948348) are investigating the impact of ICIs in combination, such as nivolumab and ipilimumab or pembrolizumab, in dMMR tumors. These studies aim to improve surgical outcomes and reduce recurrence rates, with preliminary results showing pathological complete response (pCR) rates of 60%. Additionally, ongoing trials such as ATOMIC (NCT02912559) and POLEM (NCT03827044) are evaluating ICIs combined with chemotherapy or as standalone therapy in resected stage III dMMR tumors, with the goal of improving disease-free survival. While initial results are promising, further controlled studies are needed to confirm the effectiveness of neoadjuvant immunotherapy in CRC and to establish its potential compared to conventional therapies [108].

Promising results have also been observed following endoscopic resection of CRC in these patients, particularly those with high MSI-H or dMMR. In a retrospective study conducted by Fox et al., which analyzed localized dMMR CRC in 38 patients, 45% achieved a complete endoscopic response, and 23% achieved a radiological response after four cycles of treatment. Additionally, 61% of patients who underwent surgical resection following neoadjuvant therapy achieved a pCR [109]. In another study evaluating the utility of PD-1 inhibitors in these patients, the response of polyps was also assessed. Among 26 patients with polyps, seven adenomas disappeared following treatment, all of which were >7 mm in size [110]. This phenomenon may be explained by a specific immunogenic profile of these polyps, characterized by higher levels of pathogenic variants and neoantigens [111]. While these findings are promising, further studies are needed to validate these results and to identify which patients may benefit most from these treatments.

The safety profile of PD-1 inhibitors is also superior to that of chemotherapy. The KEYNOTE-177 trial demonstrated that grade 3 or higher adverse events related to pembrolizumab occurred in 22% of patients, compared to 66% in the chemotherapy group [106] Similarly, the CHECKMATE-142 trial, which analyzed grade 3 or higher adverse events related to nivolumab treatment, showed a 22% incidence compared to 48% in patients treated with chemotherapy [104]. These findings not only highlight the superior efficacy of ICIs compared to conventional chemotherapy but also demonstrate a more favorable safety profile.

### Gastric cancer

Patients with LS have an estimated cumulative risk of developing gastric cancer by age 80 ranging from 0.7% to 13% [112]. This risk is higher in individuals with *MLH1* and *MSH2* pathogenic variants. Up to one-third of these patients have a family history of gastric cancer, which is an important consideration for their clinical follow-up [113]. Currently, there is insufficient evidence to recommend universal gastric cancer screening for all LS patients [114]. However, in certain countries such as the United States and the Netherlands, screening is recommended starting at 30–35 years of age, with periodic surveillance every 2–3 years [3,51,115]. A retrospective study by Kim et al. analyzed over 51,000 patients, including nearly 4000 with LS, and identified key risk factors for gastric cancer: male sex, *MLH1* and *MSH2* pathogenic variants, and the presence of first-degree relatives with the disease [116]. A recent study by Caspers et al. involving a Dutch cohort further identified *EPCAM* mutations, in addition to *MLH1* and *MSH2*, as significant risk factors. The study also highlighted that the highest risk occurs between the ages of 70 and 75, whereas the risk of gastric cancer before age 50 is minimal [117]. These factors may guide tailored surveillance strategies for LS patients.

Although clear evidence is lacking, clinical guidelines suggest screening for *Helicobacter pylori* in LS patients, considering it a cost-effective preventive measure [51].

### Small bowel cancer

The cumulative lifetime risk of developing small bowel cancer in LS patients varies significantly, reaching up to 12% [63]. The risk is particularly elevated in individuals with *MLH1* and *MSH2* pathogenic variants, with the duodenum and jejunum being the most affected sites [118–120]. Due to the limited evidence from available studies, routine screening for small bowel cancer is not currently recommended for LS patients [37,51,63].

## Genetic Counseling

Genetic counseling is a critical process that provides patients and their families with information regarding the risk of inheriting or transmitting a genetic predisposition to cancer. It includes guidance on molecular diagnostic options and strategies for prevention and early detection. In cases of suspected LS, referral to hereditary cancer units or specialized genetic counseling services is essential. These evaluations involve a detailed personal and family history, a cornerstone for accurately estimating hereditary cancer risk and informing personalized clinical management. Additionally, effective communication between the clinician and the molecular genetic diagnostic laboratory is crucial. Given the emotional burden often experienced by patients and families, psychological support is integral to the counseling process, which typically requires 60–90 min for thorough assessment [121].

Genetic testing for LS serves three main purposes: confirming the diagnosis in patients, determining the risk status of family members and guiding the management of affected and unaffected individuals [63,121]. Despite being the most common cause of HNPCC, LS remains underdiagnosed. Classical clinical criteria, such as those from Amsterdam and Bethesda, have limited specificity and sensitivity, particularly in detecting pathogenic variants in *MSH6* and *PMS2* [122,123]. Genetic counseling is recommended for individuals with a ≥5% risk of LS based on these models, and universal screening strategies are increasingly adopted to reduce diagnostic gaps [121].

The loss of expression of any of the *MLH1, MSH2, MSH6* or *PMS2* proteins serves as a referral criterion to a Cancer Genetic Counseling Unit and facilitates the targeted genetic diagnostic evaluation for LS [122,123]. Available diagnostic tests, including immunohistochemistry, MSI testing and multigene NGS enable the detection of LS and other CRC-related variants. Tumor tissue analysis is preferred for confirming LS, and genetic counseling should be offered to at-risk family members, starting with first-degree relatives. However, low rates of genetic counseling and testing have been reported among individuals with risk factors for LS [124].

### Genetic counseling process

Genetic counseling is a comprehensive process that begins with the evaluation of the index case, following a clear sequence of pre-test counseling, genetic testing and post-test counseling [125,126]. The first step involves pre-test counseling, where the patient’s family history is reviewed to assess the potential hereditary or familial cancer risk. A detailed pedigree is constructed, and the counselor provides information about the suspected hereditary syndrome, including its clinical, molecular and management aspects. This counseling also includes an explanation of the genetic testing process and informed consent as well as the potential outcomes, risks and benefits of testing. Once the pre-test phase is completed, genetic testing is performed. After the test results are available, post-test counseling is conducted to discuss the findings, their implications for the patient and the next steps in management (Table 2). However, no testing method is perfect, and sometimes genetic variants may go undetected even when present [3,127].

**Table 2:** Classification of gene variants in Lynch Syndrome: assessing probabilities of being pathogenic and their clinical implications

Class	Probability of being pathogenic	Clinical implications
*Benign (B)*	<0.001	Treat as if ‘no mutation detected’
*Likely benign (LB)*	0.001–0.099	No predictive testing of at-risk relatives is suggested
*Variant of unknown significance (VUS)*	0.100–0.899	Surveillance should be guided by family history and individual risk factors. Additional data may be needed for reclassification, and research testing of family members can be considered
*Likely pathogenic (LP)*	0.900–0.999	Full high-risk surveillance
*Pathogenic (P)*	>0.999	Predictive testing of at-risk relatives is suggested

**Figure 2:**
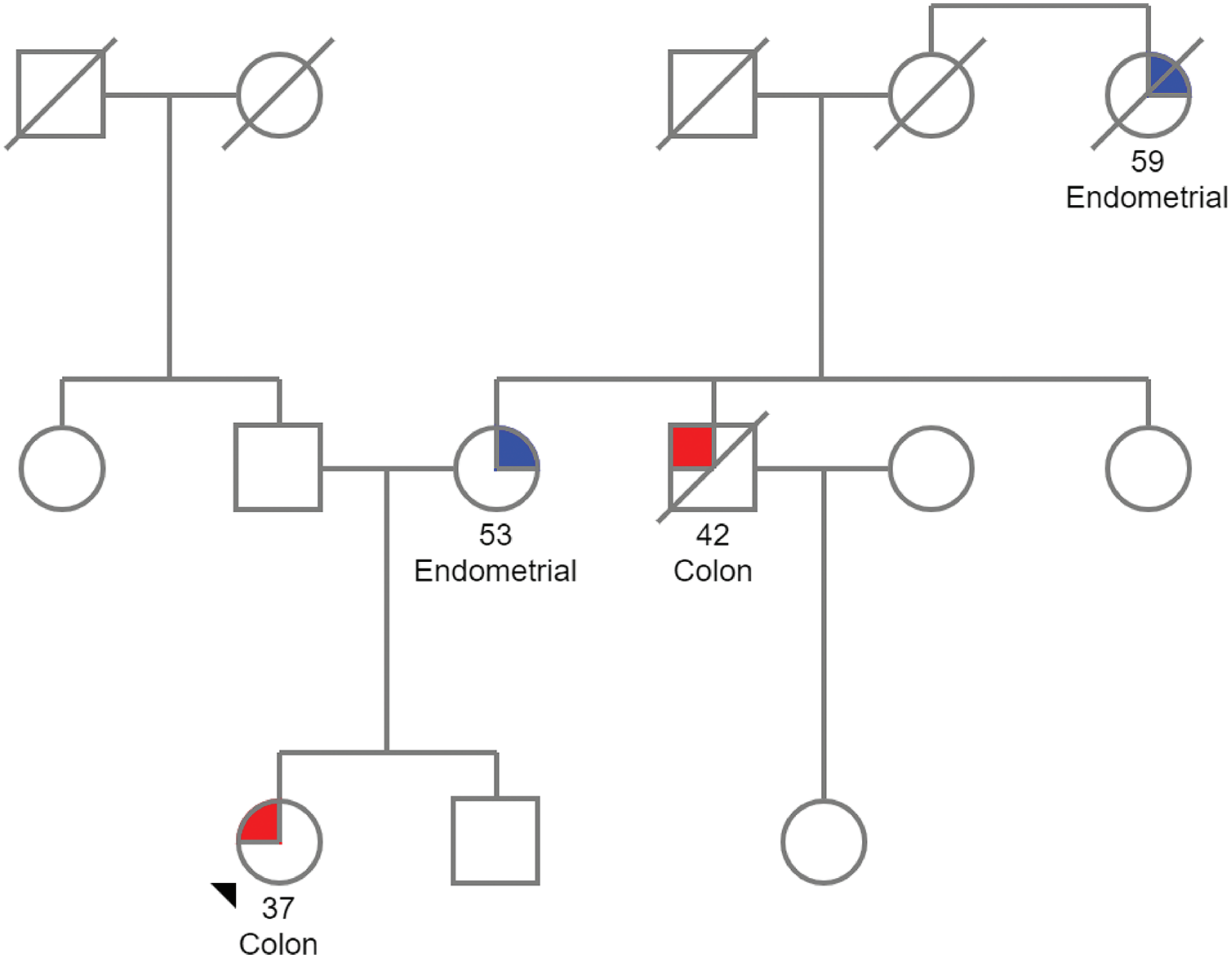
Pedigree of a family with Lynch syndrome. The proband, located at the lower left of the pedigree, is a woman diagnosed with CRC at the age of 37. Her mother was diagnosed with endometrial cancer at 53, her uncle passed away from CRC diagnosed at 42, and a maternal great-aunt died from endometrial cancer. Given this family history of LS-associated tumors, genetic counseling is recommended. The pedigree was created using the Pedigree software from Progeny Genetics.

Identifying the carrier of a germline pathogenic variant is critical for managing both the proband and their family (Fig. 2). Once LS is confirmed in the index case, genetic counseling should be offered to first-degree relatives. In this regard, while genetic testing is typically not recommended for individuals under 18, predictive testing may be considered in cases with a family history of early-onset cancers [121,122]. The same process of pre-test counseling, testing and post-test counseling is then extended to at-risk family members identified through the family pedigree. Pre-test counseling for relatives involves providing them with information about the inherited condition and the potential risks of carrying the pathogenic variant. This is followed by genetic testing, which aims to identify individuals who may benefit from early surveillance or preventive measures. Finally, post-test counseling is offered to discuss the results, the implications for the relative’s health and the need for ongoing monitoring and intervention. Several challenges can arise when taking a family history and can obscure the potential presence of a hereditary cancer syndrome, such as incomplete family structure due to unknown or adopted family members, small family size or the loss of relatives at a young age from non-cancer-related causes [127]. These limitations should be carefully considered during the assessment and counseling process. Furthermore, information must be presented clearly, considering the patient’s educational level and prior understanding of genetics.

Once the pathogenic variant responsible for the disease is identified in the index case, this enables accurate identification of true positive and true negative results in family members. A true positive occurs when the same known pathogenic variant is detected in a family member, confirming their increased risk for associated cancers and guiding the implementation of preventive measures. Conversely, a true negative arises when a family member is found to lack the pathogenic variant, confirming their cancer risk is similar to that of the general population, and no special follow-up is required. At this stage, Variants of Unknown Significance (VUS) no longer applies, as the pathogenic variant has already been conclusively identified [128]. Therefore, ensuring that affected individuals and their families understand the implications of the results and receive appropriate guidance for decision-making and management strategies is fundamental.

### Reproductive options

LS follows an autosomal dominant inheritance pattern, with a 50% chance of passing the pathogenic variant to offspring [125]. When a germline heterozygous LS-related pathogenic variant is identified in a family member, options for prenatal and preimplantation genetic testing (PGT) become available. While natural conception remains an option for individuals willing to accept this risk, these alternative reproductive strategies are available for those seeking to prevent transmission. However, the use of such testing is a subject of ongoing debate within the medical community and among affected families, as perspectives often reflect differing ethical, cultural and personal values [129,130].

Prenatal genetic testing for LS aims to determine during an ongoing pregnancy whether the fetus has inherited the pathogenic variant. Traditionally, this testing involved invasive procedures, such as chorionic villus sampling or amniocentesis, to collect fetal genetic material. However, advancements in non-invasive prenatal testing (NIPT) now allow analysis using a maternal blood sample, reducing the physical risk to the fetus [131,132]. These molecular diagnostic approaches, supported by technologies like NGS, have made testing more accessible and precise. Nevertheless, selecting the appropriate method for each case is critical, requiring an understanding of the available techniques and their limitations to ensure accurate results.

Otherwise, in recent years PGT offers an alternative during *in vitro* fertilization cycles, allowing screening of embryos at the blastocyst stage to identify those free of the LS pathogenic variants [133–135]. Selected embryos are implanted, minimizing the risk of transmission. Although PGT has increased in priority due to its ability to provide greater reproductive control, it is still constrained. This is due to the substantial medical, financial and emotional investment required, as well as the need for specialized facilities [129,135]. Additionally, the use of donor gametes represents another viable option for at-risk families.

Due to the high risk of infertility associated with LS, either as a result of the disease itself or the medical or surgical treatments that may affect germ cells, it is recommended to inform patients about fertility preservation options at the time of diagnosis [129]. For women with reduced ovarian reserve or those who have not pursued fertility preservation procedures, egg donation presents a viable reproductive option. Additionally, discussions with women with LS should include the option of risk-reducing surgeries, such as hysterectomy and bilateral salpingo-oophorectomy, which are typically recommended after childbearing or by age 40 to mitigate the elevated risk of gynecological cancers [136].

It is worth noting that prenatal diagnosis for LS presents unique challenges, as it involves a condition with adult onset, incomplete penetrance and variable expressivity. Unlike conditions traditionally considered for prenatal testing—typically severe, early-onset and highly penetrant—LS poses a complex and ethically sensitive scenario due to the significant variability in cancer risk based on the specific gene pathogenic variant and other modifying factors [137–140]. Moreover, these current approaches highlight the need for advanced molecular technologies and informed counseling to guide decision-making in LS management.

In cases of mosaic LS, genetic counseling is essential for discussing its implications on offspring. Although rare, the risk of passing on the variant may be lower in mosaic cases, but careful evaluation and reproductive planning with a genetic counselor are still necessary. PGT-M can be considered to prevent the transmission of LS to offspring [134]. Additionally, it presents unique challenges in patient management. It results from a post-zygotic mutation, meaning that the pathogenic MMR gene variant is present only in a subset of cells. This leads to variable expression of the syndrome, complicating both genetic testing and cancer risk assessment. Surveillance strategies may need to be tailored to each patient, as the frequency and intensity of monitoring, such as colonoscopies, may differ depending on the extent of mosaicism [57,141]. Furthermore, the cancer risk in mosaic LS cases may be lower or more unpredictable compared to germline genetic variants, requiring a personalized approach to risk management.

## Conclusions

LS is characterized by pathogenic variants in MMR genes, with *MLH1* and *MSH2* variants presenting a higher lifetime risk of CRC compared to *MSH6* and *PMS2*. The variability in cancer risk associated with MMR genes has significant clinical implications. Surveillance strategies should be tailored to reflect gene-specific risks, prioritizing intensive monitoring for *MLH1* and *MSH2* carriers while considering less frequent screening for *MSH6* and *PMS2* carriers. Moreover, the observed differences in penetrance between PLSD and IMRC cohorts suggest regional or methodological variations that merit further investigation. Further studies are needed to refine risk stratification and improve management protocols, particularly for underrepresented groups such as *PMS2* carriers.

Colonoscopy remain the primary method for CRC prevention in LS patients, with screening beginning at age 25 for *MLH1* and *MSH2* carriers, and at age 35 for *MSH6* and *PMS2* carriers. Adherence to screening protocols is critical to reduce CRC incidence, emphasizing patient compliance and high-quality colonoscopies. Despite advancements in genetic testing, LS remains underdiagnosed, as current clinical criteria like the Amsterdam and Bethesda guidelines show reduced sensitivity in detecting *MSH6* and *PMS2* pathogenic variants. This highlights the need for more inclusive testing approaches. Universal screening strategies are recommended to address these gaps and enable earlier detection of LS. Furthermore, an improved understanding of gene-specific risk differences has led to more personalized clinical management recommendations.

Genetic counseling is crucial for managing LS, offering patients and families essential information on genetic risk, inheritance and personalized care. However, its practice remains controversial and is influenced by factors such as institutions, geographic region and healthcare policies, leading to inconsistencies in accessibility and quality. To improve LS management, further research is needed to refine risk stratification, enhance genetic testing (e.g., NGS) and enhance surveillance protocols. Additionally, the integration of artificial intelligence in colonoscopy and gene-specific interventions could optimize care. Raising awareness and education among healthcare providers are essential to promoting genetic evaluation and testing uptake, ensuring that patients receive timely and informed guidance to improve outcomes and management of LS.

## Data Availability

Data sharing not applicable to this article as no datasets were generated or analyzed during the current study.
